# Determination of Glucose Concentration in Aqueous Solution Using ATR-WT-IR Technique

**DOI:** 10.3390/s90806254

**Published:** 2009-08-06

**Authors:** Samer Ibrahim Al-Gharabli

**Affiliations:** Chemical-Pharmaceutical Engineering Department, School of Applied Medical Sciences, German-Jordanian University, P.O. Box 35247 Amman 11180 Jordan; E-Mail: samer.gharabli@gju.edu.jo; Tel.: +9262-6-530-0666; Fax: +9262-6-530-672

**Keywords:** diabetes, biosensors, glucose monitoring, wavelet, neural network, noninvasive sensing, infrared, ATR

## Abstract

Fourier transformation infrared (FT-IR) spectroscopy has been used to measure glucose concentrations in different matrices. The accuracy of the FT-IR technique does not meet the requirements of medical applications, so we have developed a new, efficient and precise method based on attenuated total reflectance coupled with wavelet transformation (ATR-WT-IR). One thousand interferograms, divided into training- and testing-sets, have been recorded from four glucose concentrations using an ATR-IR unit. Signals were subjected to (WT) and neural network (NN) study in order to design correlation algorithm. The Pearson’s Correlation Coefficient (PCC) obtained by judging the predicted- against the real-concentrations was 0.9954, with a mean square error of 8.4e-005. The proposed ATR-WT-IR method shows efficiency in glucose prediction and could possibly to be integrated into a non-invasive monitoring technique.

## 1. Introduction

Diabetes is a chronic disease that results from deficiency in insulin production or the presence of ineffective insulin. This will lead to uncontrolled amounts of glucose in blood. Control of sugar levels in blood is essential for diabetes mellitus patients to prevent enormous hazardous side effects [1]. Improved technology for measuring blood glucose is rapidly changing the standards of health care. Currently, there are several methods for testing blood glucose levels. All of these methods are expensive and require a finger puncture, that is relatively unsafe and painful, especially if repeated many times. Patient’s sensitivity to finger punctures, possible infections and the relative handling difficulty by patients urge the development of noninvasive techniques [2].

Although the cost of glucose monitoring tests seems high, it is still incomparable with the costs of diabetes complications. The development of techniques to monitor glucose in serum is challenging due to the complicated serum matrix, which includes several types of proteins, sugars, lipids, small molecules, as well as varied concentrations of nutrients [3–5].

In this context, infrared spectroscopy (IR) emerges as a powerful methodology. Nevertheless, for medical requirements, only reliable methods with high accuracy and precision are acceptable [6–10]. IR signals reflect selectively the concentration of glucose in blood without exposing patients to dangerous radiation or to direct penetration of their skin. However, peak broadening or overlapping with signals from other constituents will diminish the selectivity. The traditional Fourier Transformation (FT) of IR signal does not effectively distinguish matrix signals. In this work, a novel signal transformation was applied by decomposing IR signals according to the Wavelet Transformation (WT) technique. Data were collected using attenuated a total reflectance (ATR) technique and subjected to a neural network (NN) study for concentration prediction.

## 2. Materials and Methods

### Sample Preparation

Glucose was purchased from Sigma-Aldrich (Ingelheim/Germany). IR spectra were recorded on TENSOR Series ATR FT-IR Spectrometer (Bruker/Germany). Aqueous solutions of 0.05, 0.10, 0.20, and 0.40 M glucose were prepared in Milli-Q water.

### Wavelet Transformation

Wavelets and wavelet transforms are relatively new powerful tools for hierarchical multi-resolution analysis of signals. They have been widely applied to a diverse fields, including signal processing, physics, astronomy, medical imaging, and image processing. Wavelets have achieved great success in image compression, enhancements, analysis, classifications, and retrieval.

Wavelets $\psi_{k}^{j}$ are basis functions, derived from scaling and translation of a single mother wavelet function *ψ* that represent a signal f(x) in different frequency bands, each with a resolution matching its scale. Thus, those wavelets are called space-scale wavelets and are used for multi-resolution decomposition of a signal. The word scale refers to the different resolutions of the wavelet transformed signal. Wavelets are defined over a finite interval and having an average value of zero. Wavelets can be expressed by the following equation:

$$\psi_{k}^{j}\;(x)=\frac{1}{\sqrt{2^{j}}}\psi \left(\frac{x}{2^{j}}-k\right)$$

where, j is the scale (or level) and k is the translation (or location). The wavelet decomposition of a signal f(x) is given using this function:

$$w_{k}^{j}=\int f(x)\psi_{k}^{j}\;(x)\mathrm{dx}=\frac{1}{\sqrt{2^{j}}}\int f(x)\psi \left(\frac{x}{2^{j}}-k\right)\mathrm{dx}$$

where $w_{k}^{j}$ are called wavelet coefficients that make the wavelet domain representation of the function f(x). It is important to note that the wavelet coefficient represents the degree of correlation (or similarity) between the function and the mother wavelet at the particular scale and transition. The reconstruction of the signal f(x) is performed by the inverse wavelet transform, namely:

$$f(x)=\sum_{j,k}w_{k}^{j}\;\psi_{k}^{j}\;(x)=\sum_{j,k}\frac{1}{\sqrt{2^{j}}}\;w_{k}^{j}\psi \left(\frac{x}{2^{j}}-k\right)$$

## 3. Results and Discussion

The signal transformation process takes place in a multi level decomposition. At the first level the signal is decomposed into approximation and detailed coefficients, for the second level the approximation coefficients decomposed again into approximation and detailed coefficients, the decomposition process iterated until reach the pre determined levels number.

**Figure 1.**
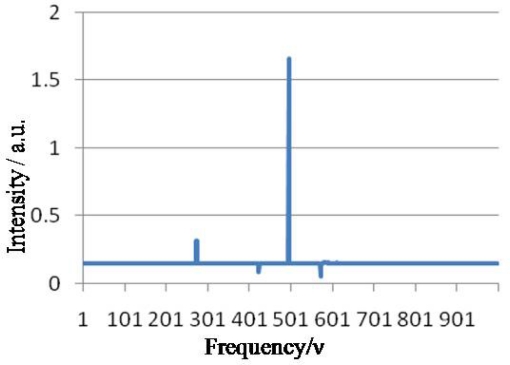
WT transformation of IR-ATR signals of a glucose solution.

In this work as an example, four glucose concentrations were measured 250 times each using IR-ATR technique. The resulting interferogram signals were transformed by the WT algorithm. As it is shown in Figure 1, the spectrum that results from use of this technique shows enhanced selectivity and resolution.

**Figure 2.**
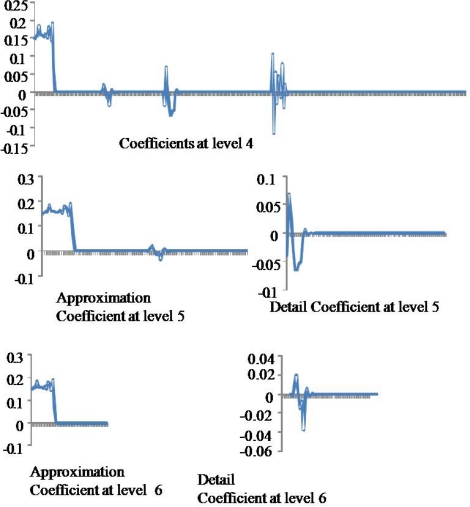
Three levels in the Wavelet Decomposition of IR-ATR signals.

For further signal transformation analysis, the technique use the detail and approximation coefficients at each level (Figure 2). Starting with the approximation coefficients at the final level and adding the detail coefficients for each upcoming level to predict the relation between the coefficients and the accuracy of the technique. There are a number of wavelet functions that can be used as a mother for WT. Therefore, the details of the particular application should be taken into account and the appropriate function should be chosen in order to use the Wavelet Transform effectively. In this approach, we make use of Daubechies wavelets family and Daubechie 9 as a wavelet function with wavedec procedure of six-levels. Signals were decomposed downstream using six levels and resulting data were analyzed using neural network (NN) technique for the best correlation between signal and concentration.

**Figure 3.**
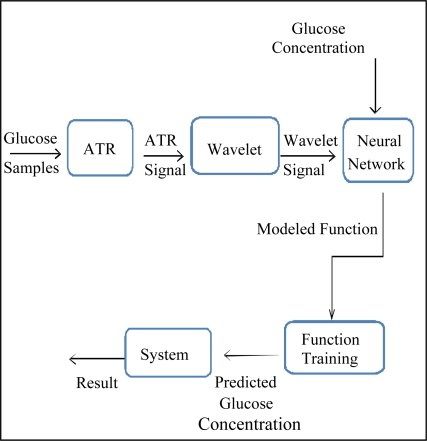
Architecture of data analysis and treatment.

The overall architecture of the work is shown in Figure 3. The input for the NN consists of 1,000 sequences, where each sequence represents the wavelet values, and the output was the concentration of the corresponding sequences. The values of the glucose concentration were 0.05, 0.1, 0.2, and 0 .4 M with the same percentage of signals (25%). The neural network employed here is feed forward back propagation NN with the trainlm training function, and learngdm adaption learning function. The learning rate and momentum was adjusted at 0.001 and 0.4, respectively. Using 100 hidden layers, in addition, weights are initially generated by random number generators and iteratively optimized to maximize the Pearson’s Correlation Coefficient (PCC) between predicted and actual value of the concentration. In the training process the training-, validation- and testing- sets are selected randomly as 60% of the data for training, 20% for testing, and 20% for validation. The final result is assessed by (PCC) and Mean Square Error (MSE). MSE is the absolute difference between predicted and actual values of glucose concentration.

Iterations for learning stop if the goal of the best PCC is met or the number of iterations (epochs) is reached. The work is implemented into two stages, in the first stage the influence of the wavelet coefficients are examined by training the NN with 100 epochs using the approximation coefficient at level six, after that, adding the detailed coefficient for the next upcoming levels. The second stage is to inspect the influence of the number of epochs in predicting the accuracy of the concentration.

**Figure 4.**
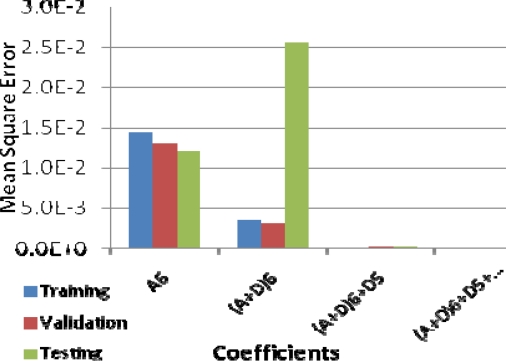
Mean Square Error (MSE) for different set of coefficients.

**Figure 5.**
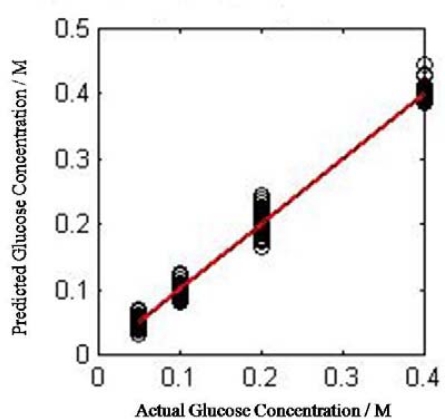
The correlation coefficient between the actual and predicted glucose concentration.

The NN is trained first to predict the influence of the approximation and detailed coefficients in representing the original signal and its relation with the glucose concentration. The correlation coefficient is increased and the MSE is decreased when adding the detailed coefficient at each step. PCC using approximation coefficient for testing data at level six was 0.5230756, when adding the detail coefficient for the same level it became 0.558089, and 0.99575 when adding detail coefficient from level five. The MSE was 1.686e-4 and 1.215e-2 at level five and six respectively (Figure 4). Therefore, it is a monotonic increasing in the PCC when adding the coefficients at each level and in the same manner monotonic decreasing in the MSE for the same set of coefficients. In the next stage, the approximation coefficients at the six-level were used. At the beginning the MSE goal for the training is set to zero and the number of iterations is set to 10,000, the resulting PCC was 0.9615. We notice that the performance of the training can be increased if the number of the iterations is increased, so we attempted to increase the number of iterations with the same previous parameters for the NN and finally obtained better prediction accuracy with PCC equal to 0.9956 and MSE 8.4e-005. The training process stopped when the performance increased slowly or the number of iteration is met. Figure 5, shows the Pearson’s correlation coefficient between the actual glucose concentration and the predicted one. The correlation coefficient using the approximation coefficient at the last level was increased from 0.5230756 to 0.9954 when the iteration number increased.

## 4. Conclusions

In this work, wavelet transformation has been implemented for ATR-IR signal transformation. A neural network has been constructed and used for concentration prediction where the correlation coefficient between the two actual and predicted concentrations was 0.9954. Therefore, ATR-IR coupled with wavelet transformation would be a promising tool to increase the accuracy of non-invasive glucose monitoring devices.
